# A Comparison of Clinical Outcomes Between Laparoscopic and Open Abdominal Myomectomy in Women With Multiple Symptomatic Uterine Fibroids: A Systematic Review and Meta-Analysis

**DOI:** 10.7759/cureus.97211

**Published:** 2025-11-19

**Authors:** Shamaila Ibrahim, Bijendra Patel, Md. Rezaul Karim

**Affiliations:** 1 Obstetrics and Gynecology, King's College Hospital, London, GBR; 2 Barts Cancer Institute, Queen Mary University of London, London, GBR; 3 Obstetrics and Gynecology, Homerton Healthcare NHS Trust, London, GBR; 4 Surgical Science, Barts Cancer Institute, Queen Mary University of London, London, GBR; 5 Surgery, The Royal London Hospital, London, GBR

**Keywords:** fibroid uterus, fibroma or fibromyoma, infertility, laparoscopy* or minimally invasive or keyhole and open or laparotomy and myomectomy or fibroidectomy and myoma or fibroid or leiomyoma, leiomyoma, open myomectomy or open abdominal myomectomy, or laparotomy myomectomy, or pregnancy

## Abstract

Uterine fibroids are the most common benign tumors of the uterus, affecting millions of women worldwide. Laparoscopic myomectomy (LM) and open myomectomy (OM) are the two most common surgical modalities for treating uterine fibroids, particularly in women wishing to preserve their uterus. This systematic review and meta-analysis aims to compare intraoperative, postoperative, and obstetric outcomes of LM and OM in women with uterine fibroids. Adhering to PRISMA (Preferred Reporting Items for Systematic Reviews and Meta-Analyses) guidelines, a comprehensive literature search was performed using PubMed, Embase, Web of Science, Scopus, Cochrane, Ovid platform, and Google Scholar. Data analysis was conducted using an Excel spreadsheet, and meta-analysis was performed on Review Manager (RevMan) version 5.4.1 (The Cochrane Collaboration, London, UK). Results were presented in the form of mean and standard deviation (SD), standardized mean difference (SMD), 95% confidence interval (CI), and risk ratio (RR). Heterogeneity (I²) was considered significant when it exceeded 50%. For the quality assessment of the risk of bias, ROBINS-I and RoB 2 tools were used. GRADEpro was used to generate the summary of findings tables for primary and secondary outcomes. Analysis was conducted on 14 published studies, comprising 12 non-randomized trials, mainly retrospective, and 2 randomized trials. The review included studies from 12 different countries, spanning from 2000 to 2024, involving a total of 3,828 women with uterine fibroids (1,879 LM, 1,949 OM). The age of participants ranged from 18 to 50 years (mean age: LM, 39.82 ± 4.02; OM, 35.61 ± 3.83). Key findings indicated that LM resulted in significantly less blood loss than OM (SMD 1.21), shorter hospital stay (SMD 1.76), and less postoperative hemoglobin drop (SMD 1.44). However, LM had a longer duration of surgery (MD = 12.29 minutes, *P* = 0.20). Both the number and size of myomas removed are slightly lower in LM compared to OM, with statistically significant differences (*P* < 0.0001); however, high heterogeneity across studies suggests notable variability in outcomes. Obstetric outcomes favored LM with a higher pregnancy rate (29.30% vs. 22.40%, *P* = 0.01) and a higher rate of normal vaginal delivery (37.90% vs. 32.10%, *P* = 0.27), and a lower caesarean section rate (47.40% vs. 57.80%, *P* = 0.17) among women who conceived post-myomectomy as compared to OM. No significant difference was observed in miscarriage rates (13.50% vs. 14.10%, *P* = 0.90) between the two groups. In conclusion, LM offers significant advantages in reducing intraoperative and postoperative morbidity compared to the traditional open approach. However, determining the superior approach in terms of minimizing complications and improving obstetric and long-term fertility outcomes remains a subject of ongoing debate. The choice of procedure depends on surgical expertise and patient-specific factors: number of fibroids, site, and size of fibroids. The laparoscopic approach for multiple myomas is still controversial, while open myomectomy appears more suitable for multiple and large-sized fibroids.

## Introduction and background

Uterine fibroids (UFs) are the most common benign tumors of the female genital tract [1,2], originating from smooth muscle cells and fibroblasts of the uterine myometrium, and they are a leading cause of heavy menstrual bleeding in women of reproductive age. Fibroids may be single or multiple, vary in size, and may develop anywhere within the uterine wall. Based on location, they are classified as subserosal (on the outer uterine surface), intramural (within the uterine muscle layer), submucosal (beneath the inner lining), or pedunculated (protruding outside the uterus on a stalk) lesions. The incidence of UFs varies by ethnicity and age. By age 35, fibroids occur in 60% of African-American women, increasing to 80% by age 50, compared with 40% and 70% among Caucasian women [1]. Prevalence ranges from 4.5% to 68.6%, depending on age and ethnicity [2]. Although 50%-75% of UFs are asymptomatic, about 30% of women experience symptoms depending on fibroid size, number, and location. Common symptoms include abnormal uterine bleeding, menorrhagia, and anaemia [1,2]. Large or multiple fibroids may cause pressure symptoms such as urinary frequency, dysuria, constipation, pelvic pain, and subfertility [3]. Women of African origin in Europe tend to have more severe symptoms, earlier surgery, and a recurrence rate of 59% within 4-5 years post-myomectomy [4]. Fibroids are found in 5%-10% of infertile women, with 2%-3% of cases solely attributed to fibroids [5]. The prevalence of fibroids during pregnancy is 10.7% in the first trimester, with 10%-30% experiencing adverse obstetric outcomes. Complications are more frequent when fibroids exceed 200 cm³ [6].

Laparoscopic myomectomy (LM) is a minimally invasive procedure to remove fibroids while preserving the uterus. It involves a uterine manipulator and trocars placed based on uterine size and fibroid location. Closed or open-entry techniques may be used depending on the surgeon's discretion [7]. LM offers reduced blood loss, less pain, shorter hospital stays, fewer infections, faster recovery, and earlier return to daily activities. Limitations include difficulty with multiple or large fibroids (>10 cm), which may increase surgical complexity and time. Fibroids are also linked to increased risk of placenta previa (placenta covering the cervix) and post-operative adhesion formation, which may cause pain or infertility. In LM, inability to palpate the uterus may lead to missed intramural fibroids (within the uterine muscle layer), which can be mitigated by preoperative or intraoperative ultrasound [8].

Open abdominal myomectomy (OM) involves a larger incision to access and remove fibroids. The uterine wall is incised, fibroids are enucleated, and the defect is sutured to restore integrity. OM allows tactile identification and direct palpation, making it suitable for multiple or deeply located UFs. However, it involves greater blood loss, longer hospital stays, more postoperative pain, a higher rate of infection and adhesion formation compared to LM [9].

This comparison is particularly relevant in global contexts where surgical expertise, training, and access to minimally invasive equipment vary. Understanding the relative benefits of LM and OM can inform clinical decision-making in both high-resource and resource-limited settings.

Critical literature review

Several studies and reviews have compared LM and OM for efficacy, safety, and reproductive outcomes. A systematic review by Cianci et al. [10] concluded that the critical factor influencing the choice of surgical approach is primarily the size and quantity of fibroids, with mini-laparotomy being a viable intermediate option. The type of surgical procedure appears to have no significant impact on the pregnancy rate [10]. A meta-analysis [11] reported that LM seems to be a better choice for patients with UFs. However, additional studies are needed to clarify the roles of LM and OM in the treatment of UFs and to assess cost-effectiveness and long-term outcomes [11]. A Cochrane review [12] found no clear superiority among surgical techniques. There is limited evidence to determine the role of myomectomy for infertility in women with fibroids, as only one trial compared myomectomy with non-surgical management. If the decision is made to have a myomectomy, the current evidence does not indicate a superior method (laparoscopy, laparotomy, or different electrosurgical systems) to improve rates of live birth, preterm delivery, clinical pregnancy, ongoing pregnancy, miscarriage, or caesarean section [12]. A study by Devassy et al. [13] observed that contained electromechanical morcellation has emerged as a safe approach for laparoscopic myoma tissue retrieval. This large series demonstrates that laparoscopic bag morcellation is a safe and comfortable method to remove large and giant uterine tumors. Bag manipulation takes only a few minutes; perforations rarely occur and are easy to detect intraoperatively. This technique did not result in the spread of debris during myoma surgery, potentially avoiding the additional risk of parasitic fibroma or peritoneal sarcoma [13]. Lu et al., in a retrospective cohort study, concluded that the size of myoma may influence the pregnancy rate of patients after LM. The number of fibroids can affect the incidence of placental adhesions during postoperative pregnancy [14]. Collectively, LM offers significant perioperative and recovery advantages over OM, especially for limited fibroid size and number. 

Knowledge gap and research significance 

For many years, OM has been the treatment of choice for symptomatic multiple myomas; however, the benefits of the laparoscopic approach in such women remain controversial. More prospective studies are required to observe surgical, obstetric, and future fertility outcomes following LM for the management of multiple UFs. 

Research question

Is LM more beneficial compared to OM in terms of outcomes and advantages for women with multiple symptomatic myomas?

Objectives

This systematic review and meta-analysis aimed to compare the clinical outcomes of LM versus OM in women of reproductive age presenting with symptomatic UFs. The primary objective was to evaluate clinical efficacy by assessing intraoperative blood loss, duration of surgery, number of myomas removed, and postoperative pregnancy rates. Secondary objectives focused on short- and long-term reproductive outcomes, including length of hospital stay, size of the largest myoma removed, postoperative hemoglobin drop, risk of miscarriage, and mode of delivery among women who conceived following myomectomy.

## Review

Materials and methods

Study Design

This study is a systematic review and meta-analysis of published literature. The methodology of this secondary research review was conducted according to the PRISMA (Preferred Reporting Items for Systematic Reviews and Meta-Analyses) guidelines. It comprises research based on previously published studies, including randomized controlled trials (RCTs), retrospective studies, and cohort studies comparing clinical outcomes of LM and OM. As this review does not involve direct human or animal participants and relies solely on publicly available data, formal ethical approval was not required. All included studies had obtained appropriate ethical clearance from their respective institutions, and their data were used in accordance with accepted academic standards.

Study Protocol

This systematic review was conducted by Shamaila Ibrahim as part of her MSc in Laparoscopic Surgery and Surgical Skills at Queen Mary University of London, Barts Cancer Institute.

Data Collection and Search Strategy

Data for this systematic review were retrieved from the Queen Mary University Library database between November 2023 and February 2024. Primary sources for data collection on laparoscopic versus abdominal myomectomy included PubMed, Embase, Web of Science, Ovid, and the Cochrane Library. Secondary sources included Scopus and Google Scholar.

Boolean operators such as AND, OR, NOT, and truncation symbols (e.g., *) were used in combination with relevant keywords to identify recent publications and to include all pertinent studies regardless of publication date. Filters were applied to narrow results by study type and relevance. Precise search terms included: laparoscopy, myomectomy, laparotomy, UFs, leiomyoma, infertility, and pregnancy. The following keywords were used to construct the search string to identify relevant studies: Laparoscopy* OR minimally invasive OR keyhole AND open OR laparotomy and myomectomy OR fibroidectomy AND myoma OR fibroid OR leiomyoma OR fibroma OR fibromyoma AND fertility OR infertility OR pregnancy.

PICOS framework: Inclusion and exclusion criteria 

The PICOS approach was employed to define eligibility through structured inclusion and exclusion criteria.

Table 1 presents the study selection criteria using the PICOS framework, outlining inclusion and exclusion parameters for women with symptomatic UFs undergoing LM versus OM.

**Table 1:** PICOS framework with inclusion and exclusion criteria. LM, laparoscopic myomectomy; OM, open myomectomy

PICOS	Inclusion criteria	Exclusion criteria
Population	Women with uterine fibroids/Age 18-50 years/Symptomatic fibroids/Multiple fibroids	Teenagers with fibroids/Post-menopausal women with fibroids/Asymptomatic fibroids/Sarcoma/Parasitic leiomyomas
Intervention	Laparoscopic myomectomy	Robotic myomectomy/hysteroscopic myomectomy/gasless laparoscopic
Comparator/Control	Open myomectomy	Mini laparotomy/Hysterectomy/Vaginal myomectomy
Outcomes	Intraoperative/postoperative/obstetric	Medical treatment of fibroids/Uterine artery embolization for fibroids/studies not comparing LM with OM/Not relevant to outcomes
Study	Randomized controlled trials/Retrospective studies/Cohort studies/Human studies	Systematic reviews/Case series/Surveys/Studies not in English/Animal studies

Results 

Study Selection

For quality and relevance purposes, studies were thoroughly screened by the abstract and title. Out of 1,725 studies, 633 duplicates were removed. A total of 1,092 studies were screened by abstract and title, and 1,078 studies were excluded based on exclusion criteria. Finally, 14 studies [15-28] were chosen for systematic review and meta-analysis: 12 were non-RCTs and 2 were RCTs. EndNote 20 software (Clarivate Analytics, Philadelphia, PA) was used to remove duplicates, screen titles and abstracts, and manage the bibliography.

**Figure 1:**
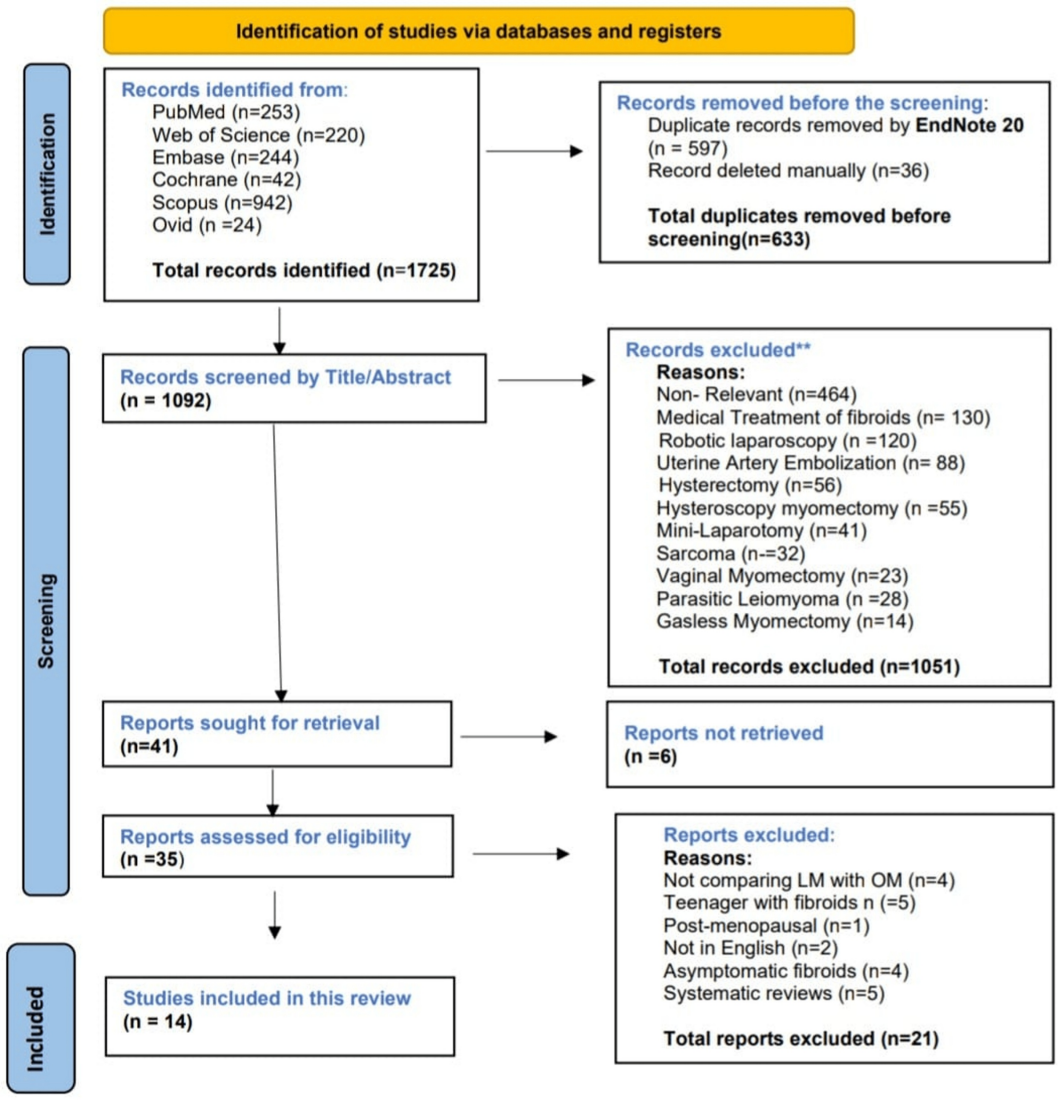
PRISMA 2020 flow diagram. Showing studies retrieved from databases, with duplicates removed in EndNote 20 and exclusions applied according to criteria. Adapted from: [29]. PRISMA, Preferred Reporting Items for Systematic Reviews and Meta-Analyses

Figure 1 shows the PRISMA 2020 flow diagram outlining study identification, screening, eligibility assessment, and inclusion.

Description of studies

The final review included 14 studies [15-28] from 12 countries, published between 2000 and 2024. Of these, 12 were non-randomized studies (mostly retrospective) and 2 were RCTs. Collectively, the studies reported outcomes for 3,828 women with UFs: 1,879 underwent LM and 1,949 underwent OM. Participant ages ranged from 18 to 50 years.

Baseline characteristics of included studies

Table 2 summarizes the baseline characteristics of the included studies comparing LM and OM. It presents details such as study design, country, duration, number of participants in each group, mean age, and intervention type.

**Table 2:** Baseline characteristics of included studies. LM, laparoscopic myomectomy; OM, open myomectomy; SD, standard deviation

Study	Year	Country	Design	Duration	LM Participants	OM Participants	Mean age LM	Mean age OM	Intervention
Seracchioli et al. [15]	2000	Italy	RCT	January 1993-January 1998	66	65	34.00 ± 4.11	33.97 ± 4.9	Experimental = LM; Control = OM
Marret et al. [16]	2004	France	Retrospective multicenter	January 1996 to January 2000	126	176	N/A	N/A	Experimental = LM; Control = OM
Holzer et al. [17]	2006	Austria	A Double-Blind Study	N/A	19	21	32 ± 7.1	32 ± 7.1	Experimental = LM; Control = OM
Chang and Chen [18]	2012	Taiwan	Retrospective study	June 2004 to October 2007	81	74	41.14 ± 6.46	40.08 ± 6.49	Experimental = LM; Control = OM
Gobern et al. [19]	2013	USA	Retrospective study	January 2007 to December 2009	73	169	39 ± 8.25	39 ± 9.25	Experimental = LM; Control = OM
Kim et al. [20]	2013	Korea	Retrospective study	January 2003 to December 2010	340	75	35.8 ± 3.4	35.2 ± 3.6	Experimental = LM; Control = OM
Kotani et al. [21]	2017	Japan	Retrospective study	January 1995 to December 2014	474	279	37.6 ± 5.2	36.0 ± 5.8	Experimental = LM; Control = OM
Gambacorti-Passerini et al. [22]	2018	Italy	Retrospective cohort study	January 2002 to December 2014	237	232	36.6 ± 4.97	N/A	Experimental = LM; Control = OM
D’Silva et al. [23]	2018	Malaysia	Retrospective cohort study	January 2010 to December 2014	67	22	33.27 ± 5.48	34.03 ± 5.42	Experimental = LM; Control = OM
Ming et al. [24]	2019	China	Multicenter cohort study	April 2012 and October 2013.-2018	83	313	31 ± 6.5	31 ± 6.5	Experimental = LM; Control = OM
Alharbi et al. [25]	2020	Saudi Arabia	Retrospective study	N/A	34	213	42.06 ± 8.72	40.82 ± 6.94	Experimental = LM; Control = OM
Kan et al. [26]	2021	China	Retrospective study	April 2016 to April 2017	42	44	N/A	N/A	Experimental = LM; Control = OM
Ordás et al. [27]	2022	2022	Retrospective cohort study	May 2012 to December 2018	112	142	34 ± 8.0	34 ± 8.0	Experimental = LM; Control = OM
Buhur [28]	2024	Turkey	Retrospective cohort study	From 2016 to 2022	84	84	36.56 ± 3.32	37.81 ± 3.13	Experimental = LM; Control = OM
Total 14 studies	2000-2024	12 countries	12 non-RCT; 2 RCT	From 2000 to 2024	Total = 1,879	Total = 1,949	LM = 39.82 ± 4.02	OM = 35.61 ± 3.83	Experimental = LM; Control = OM

Reporting quality: Risk-of-bias assessment findings

The risk of bias was evaluated using the ROBINS-I [30] tool for non-randomized studies, which were mostly retrospective, and the Rob-2 tool for RCTs.

Risk of bias of non-randomized studies by ROBINS-I

Table 3 presents the risk of bias assessment for non-randomized studies using the ROBINS-I tool, developed by the Cochrane Bias Methods Group [30]. Each study was rated as having low, moderate, serious, or critical risk of bias, with the overall judgment reflecting the highest level of bias identified across domains.

**Table 3:** Risk of bias assessed for non-RCT studies by ROBINS‑I tool. Source: [30]. ROBINS-I, Risk of Bias In Non-randomized Studies of Interventions

Author	Confounding bias	Selection bias	Bias in the classification of intervention	Bias due to deviation from the intended intervention	Bias due to missing data	Bias in the measurement of outcomes	Bias in the selection of reporting results	Overall risk
Marret et al. [16]	Moderate	Moderate	Low	Serious	Moderate	Moderate	Moderate	Serious
Chang et al. [18]	Moderate	Moderate	Moderate	Moderate	Moderate	Moderate	Moderate	Moderate
Joseph et al. [19]	Moderate	Moderate	Moderate	Moderate	Moderate	Low	Low	Moderate
Kim et al. [20]	Moderate	Moderate	Low	Moderate	Moderate	Moderate risk	Moderate	Moderate
Kotani et al. [21]	Moderate	Moderate	Moderate	Low	Moderate	Moderate	Moderate	Moderate
Passerini et al. [22]	Moderate	Moderate	Low	Moderate	Moderate	Moderate	Moderate	Moderate
D’Silva et al. [23]	Moderate	Moderate	Moderate	Serious	Lowr	Moderate	Moderate	Serious
Ming et al. [24]	Moderate	Moderate	Low	Moderate	Serious	Moderate	Moderate	Serious
Alharbi et al. [25]	Moderate	Low	Moderate	Moderate	Low	Moderate	Moderate	Moderate
Kan et al. [26]	Moderate	Moderate risk	Moderate	Moderate	Moderate	Moderate	Moderate	Moderate
Ordás et al. [27]	Moderate	Low	Low	Moderate	Moderate	Moderate	Moderate	Moderate
Buhur et al. [28]	Moderate	Low	Low	Low	Low	Moderate	Moderate	Low

**Figure 2:**
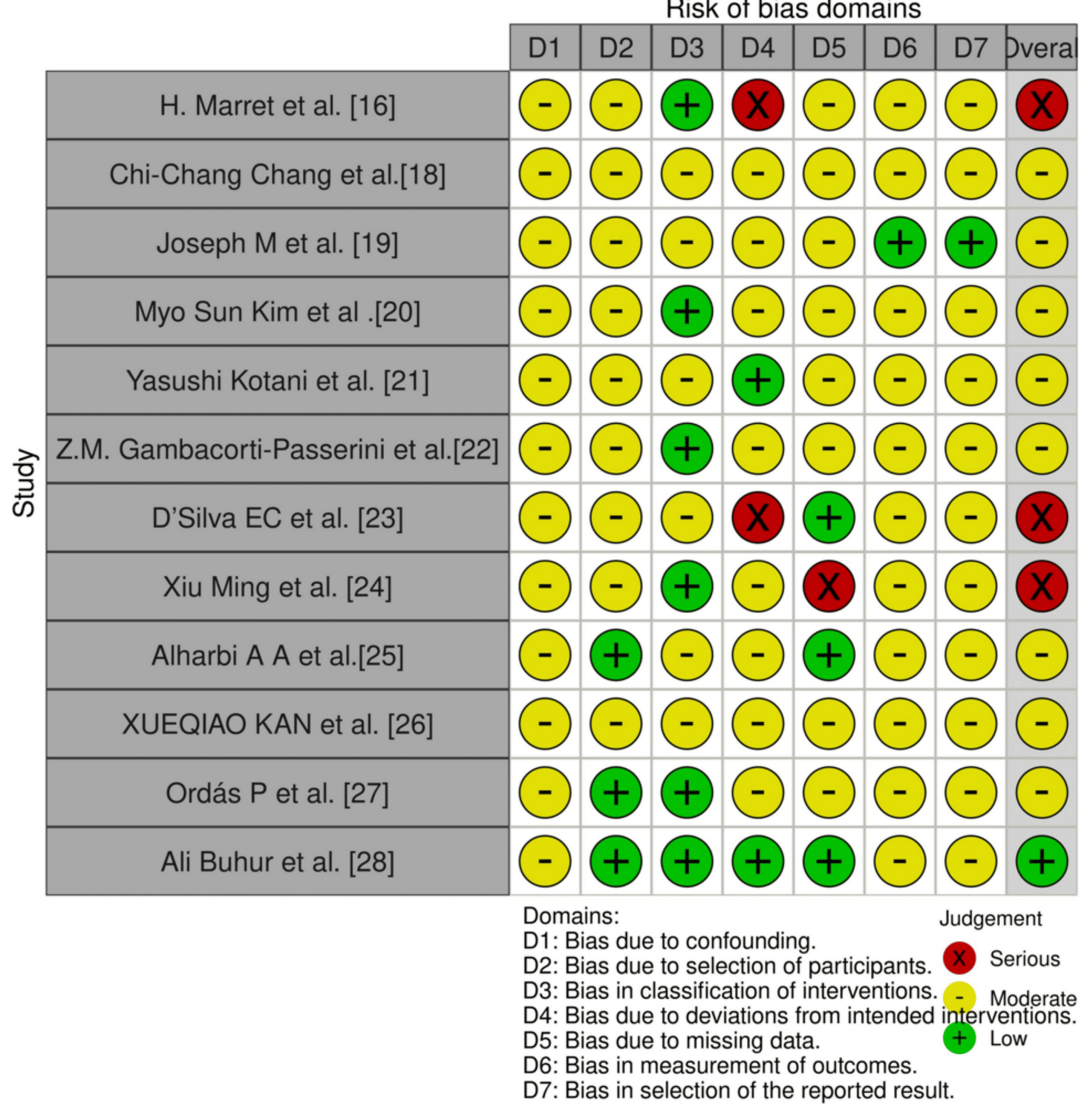
Traffic light plot for non-RCTs. The plot was generated using the Risk-of-Bias Visualization (robvis) tool. Adapted from: [31]. RCT, randomized controlled trial

Figure 2 displays a traffic light plot summarizing the risk of bias assessments for non-randomized studies, generated using the Risk-of-Bias Visualization (robvis) tool [31]. Each colored cell represents the judgment for a specific domain per study: green for *Low Risk*, yellow for *Some Concerns*, and red for *High Risk*, providing a clear visual overview of bias patterns across the included studies.

Marret et al. [16] demonstrated serious bias in the same domain, with a 29% conversion rate from laparoscopy to OM. D’Silva et al. [23] also had a serious risk of bias due to deviations from the intended intervention, as 17% of laparoscopic procedures were converted to OM. Ming et al. [24] had a serious risk of bias due to missing data, as 25% of participants were lost to follow‑up. 

Risk of bias: RCT by Rob 2

Table 4 presents the risk-of-bias assessment for RCTs using the Cochrane RoB 2 tool [32]. This tool evaluates bias across five domains: random sequence generation, allocation concealment, blinding of participants and personnel, blinding of outcome assessment, incomplete outcome data, selective reporting, and other sources of bias. Each domain was rated as *Low Risk*, *Some Concerns*, or *High Risk*, with an overall judgment reflecting the highest level of concern across domains.

**Table 4:** Risk-of-bias (RoB) assessment for randomized controlled trials by RoB 2. RoB across randomized controlled trials was evaluated and summarized using an Excel worksheet [32].

Study	Randomization process	Deviations from intended	Missing outcome data	Measurement of the outcome	Selection of the reported result	Overall
Seracchioli et al. [15]	Low	Low	Some concerns	Low	Low	Low
Holzer et al. [17]	Low	Low	Low	Low	Some concerns	Low

**Figure 3:**
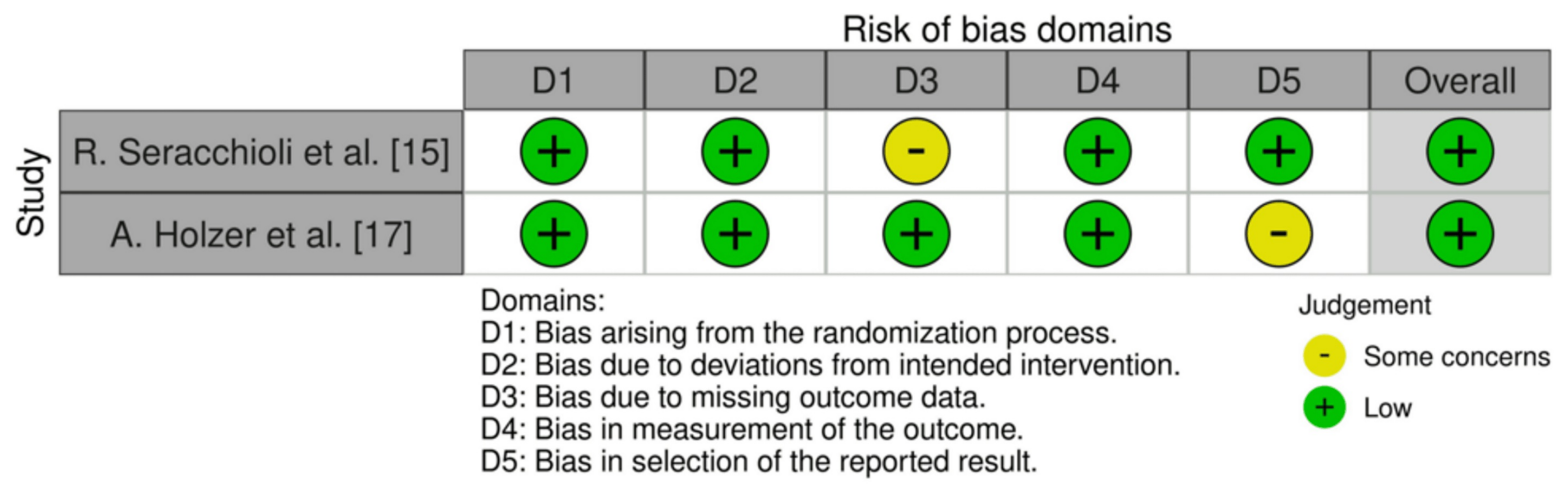
Traffic light plot for RCTs. Generated using the Risk-of-Bias Visualization (robvis) tool. Adapted from: [31]. RCT, randomized controlled trial

Figure 3 presents a traffic light plot illustrating the risk of bias assessments for RCTs, generated using the robvis visualization tool [31]. Each colored cell corresponds to a specific domain within the Cochrane RoB 2 framework: green indicates *Low Risk*, yellow denotes *Some Concerns*, and red signifies *High Risk*. This visual summary facilitates rapid comparison of bias judgments across studies and domains. 

Data extraction and analysis

Data were collected and organized using Microsoft Excel (Microsoft Corporation, Redmond, WA). Descriptive statistics, including mean, minimum, maximum, and standard deviation (SD), were calculated using the Excel Data Analysis Toolpak. Meta-analysis was performed using Review Manager (RevMan) version 5.4 (The Cochrane Collaboration, London, UK). The standard mean difference (SMD) and risk ratio (RR) were calculated with a 95% confidence interval (CI). Heterogeneity was considered significant if the I² index exceeded 50%. For continuous data, SMD was calculated; for dichotomous data, RR was estimated based on the number of events and total participants. Primary and secondary outcome data were subsequently exported from Review Manager to GRADEpro software to generate Summary of Findings (SoF) tables.

Outcome measures analyzed in the studies

Outcome measures analyzed across the included studies encompassed intraoperative blood loss, reported in 7 of 14 studies, and duration of surgery, assessed in 11 studies. The number of myomas removed and pregnancy rates after myomectomy were each documented in 8 studies. Hospital stay duration was evaluated in 8 studies, while the size of the largest myoma was reported in 9. Hemoglobin drop postoperatively was analyzed in 5 studies. Reproductive outcomes such as miscarriage, vaginal delivery (VD), and cesarean section (CS) following myomectomy were each reported in 4 studies. These measures collectively informed the comparative assessment of surgical efficacy, safety, and obstetric outcomes between LM and OM.

Primary outcomes data processing

Table 5 summarizes primary outcomes from the included studies comparing LM and OM. Not all studies reported every outcome.

**Table 5:** Primary outcomes data retrieved from included studies.

Study/Author	Blood loss (mL), LM	Blood loss (mL), LM	Duration of surgery (minutes), LM	Duration of surgery (minutes), OM	Number of removed myomas (n), LM	Number of removed myomas (n), OM	Pregnancy rate after myomectomy, LM	Pregnancy rate after myomectomy, OM
	LM (Mean ± SD)	OM (Mean ± SD)	LM (Mean ± SD)	OM (Mean ± SD)	LM (Mean ± SD)	OM (Mean ± SD)	No. of events/Total participants	No. of events/Total participants
Seracchioli et al. [15]	N/A	N/A	100.23 ± 38.34	88.85 ± 26.91	2.94 ± 2.94	2.75 ± 1.98	30/56	33/59
Marret et al. [16]	226 ± 320	504 ± 542	­­89 ± 45	89 ± 33	1.5 ± 1.5	2.9 ± 4.3	N/A	N/A
Holzer et al. [17]	71 ± 80	115 ± 64	99 ± 37	68 ± 22			N/A	N/A
Chang et al. [18]	N/A	N/A	N/A	N/A	N/A	N/A	N/A	N/A
Joseph et al. [19]	N/A	N/A	N/A	N/A	N/A	N/A	N/A	N/A
Kim et al. [20]	N/A	N/A	114 ± 96	96 ± 42	N/A	N/A	54/340	12/75
Kotani et al. [21]	207 ± 225	554 ± 536	148 ± 58	127 ± 48	3.7 ± 3.7	6.5 ± 8.6	69/474	42/279
Passerini et al. [22]	153.6 ± 150.8	284.1 ± 354.3	101.9 ± 40.8	102.2 ± 35.9	2.11 ± 2.11	2.68 ± 2.8	96/176	56/176
D’Silva et al. [23]	406.6 ± 339.55	1290 ± 1163	170.28 ± 66.7	135.73 ± 70.4			N/A	N/A
Ming et al. [24]	N/A	N/A	N/A	N/A	1.8 ± 1.8	2.37 ± 3.28	23/83	65/313
Alharbi et al. [25]	333.21 ± 392.5	576.13 ± 447.41	56.91 ± 17.91	103.05 ± 48.8	4.18 ± 4.18	3.49 ± 3.83	N/A	N/A
Kan et al. [26]	44.4 ± 5.82	79.22 ± 7.53	70.21 ± 7.83	100.23 ± 38.34	N/A	N/A	38/42	26/44
Ordás et al. [27]	N/A	N/A	147.38 ± 63.79	95 ± 37.47	1.79 ± 1.79	3.03 ± 2.86	36/112	23/142
Buhur et al. [28]	N/A	N/A	128.85 ± 36.47	79 ± 18.12	3 ± 3	3 ± 2.42	5/84	5/84
Total (participants)	962	987	1,564	1,353	1,179	1,504	1,367	1,172

Table 6 summarizes key primary outcomes comparing LM and OM. It highlights LM's advantages in reduced blood loss and higher fertility outcomes, with comparable surgical efficacy.

**Table 6:** Summary of primary outcomes data of LM vs. OM. RR, risk ratio; SMD, standard mean difference; LM, laparoscopic myomectomy; OM, open myomectomy

Outcome	Studies	Participants (LM)	Participants (OM)	Min (LM)	Min (OM)	Max (LM)	Max (OM)	Range (LM)	Range (OM)	Mean (LM)	Mean (OM)	SD (LM)	SD (OM)	No. of events (LM)	No. of events (OM)	RR	Percentage (LM)	Percentage (OM)	SMD	*P*-value
Blood loss (mL)	7	962	987	44.4	79.22	406.6	1290.5	362.2	1211.3	205.97	486.13	131.59	409.09	N/A				N/A	1.21	<0.00001
Duration of surgery (minutes)	11	1564	1353	56.91	68	170.28	135.73	113.37	67.73	111.43	98.55	34.56	19.35	N/A	N/A	N/A	N/A	N/A	12.29	0.2
Number of removed myomas (n)	8	1179	1504	1.5	2.37	4.18	6.5	2.68	4.13	2.62	3.34	0.97	1.31	N/A	N/A	N/A	N/A	N/A	N/A	N/A
Pregnancy rate after myomectomy	8	1367	1172		N/A	N/A	N/A	N/A	N/A	N/A	N/A	N/A	N/A	351	262	1.31	29.30%	22.40%	N/A	0.01

**Figure 4:**
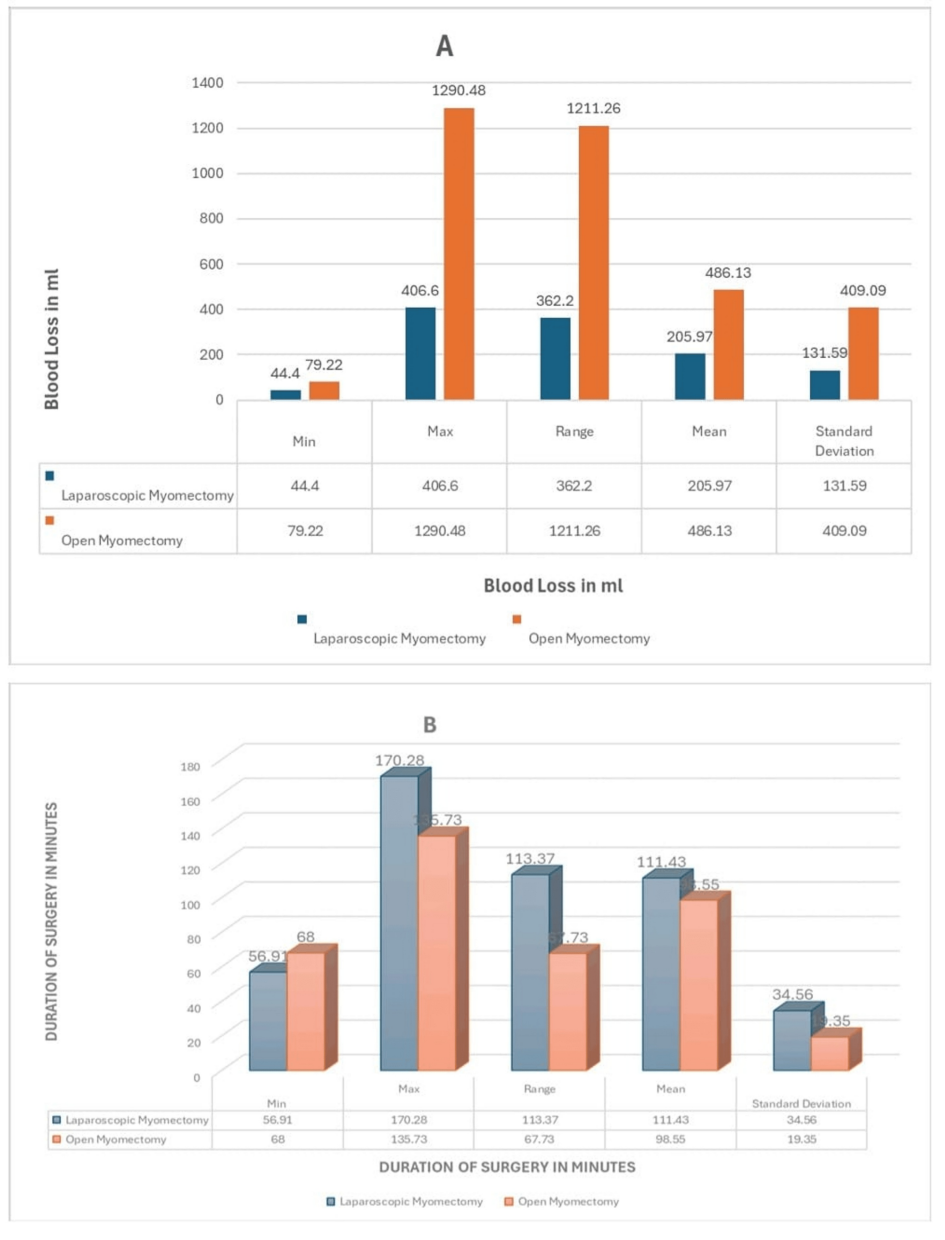
Charts of primary outcomes. (A) Blood loss (mL) in laparoscopic myomectomy (LM) vs. open myomectomy (OM), summarized by minimum, maximum, range, mean, and standard deviation. (B) Duration of surgery (minutes) in LM vs. OM, summarized by the same statistical measures.

Figure 4 presents column charts comparing LM and OM across two primary intraoperative outcomes: blood loss (mL) and duration of surgery (minutes). LM is associated with reduced blood loss but a longer operative time compared to OM.

**Figure 5:**
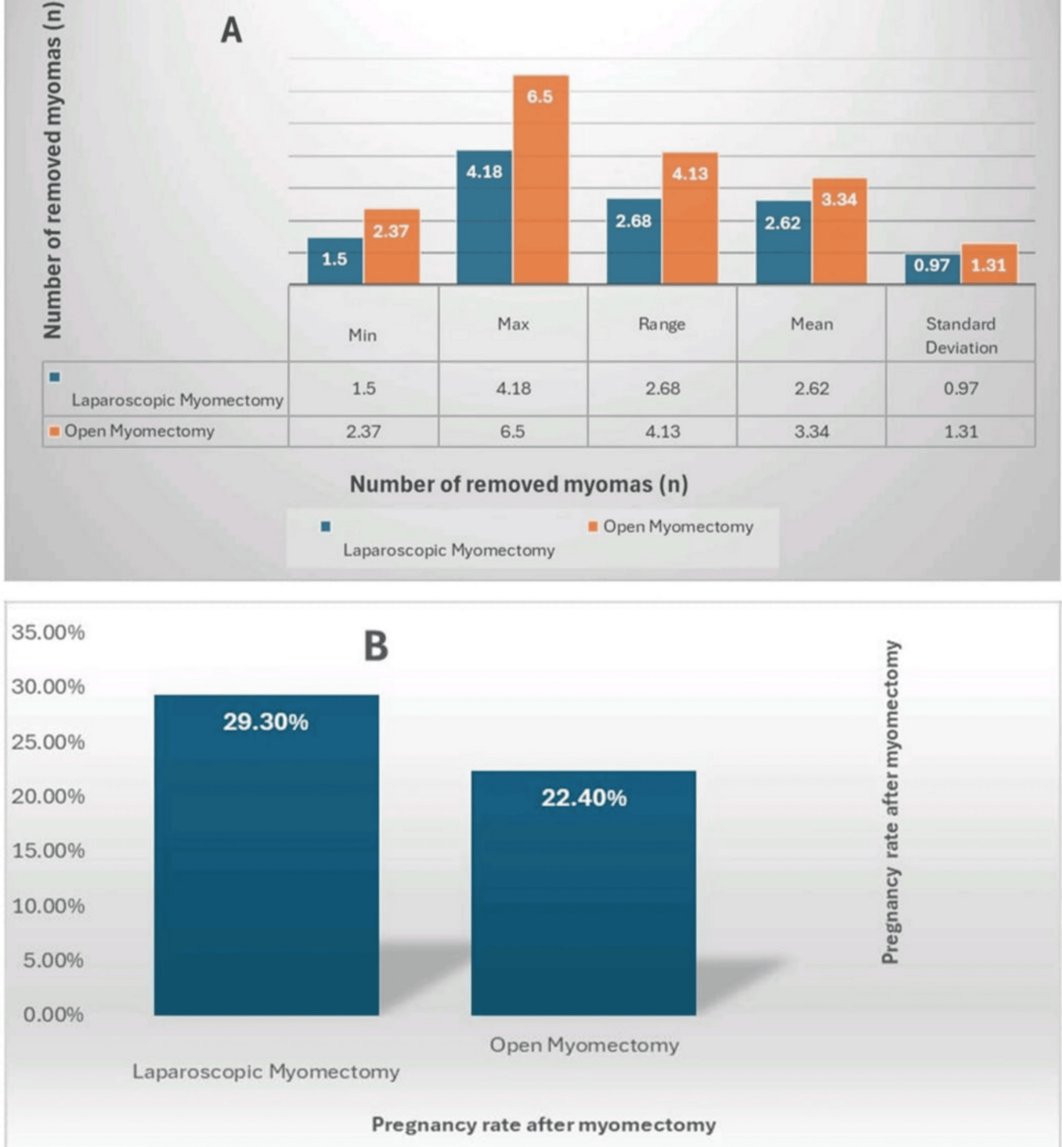
Charts of primary outcomes (continued). (A) Number of myomas removed in laparoscopic myomectomy (LM) vs. open myomectomy, summarized by minimum, maximum, range, mean, and standard deviation. (B) Pregnancy rates following myomectomy in LM vs. OM.

Figure 5 displays comparative charts for two additional primary outcomes: the number of myomas removed and postoperative pregnancy rates between LM and OM. OM is associated with a higher myoma count removal, while LM shows a slightly improved pregnancy rate. 

Table 7 presents secondary outcomes data extracted from the included studies comparing LM and OM. Continuous variables are expressed as mean ± SD, while categorical outcomes are reported as number of events. *N/A* indicates data not available or not reported in the respective study. 

**Table 7:** Secondary outcomes data retrieved from included studies.

Study/year	Hospital stay (Days)		Size of the largest myoma (cm)		Hemoglobin drop after operation (gm/dL)		Miscarriage after myomectomy		Vaginal delivery after myomectomy		Cesarean section after myomectomy	
	LM (Mean ± SD)	OM (Mean ± SD)	LM (Mean ± SD)	OM (Mean ± SD)	LM (Mean ± SD)	OM (Mean ± SD)	No. of events - LM	No. of events - OM	No. of events -LM	No. of events - OM	No. of events - LM	No. of events - OM
R. Seracchioli et al. [15]	3.15 ± 1.52	5.95 ± 1.44	7.07 ± 2.54	7.47 ± 2.6	1.33 ± 1.23	2.17 ± 1.57	06	04	07	06	13	21
Marret et al. [16]	3.6 ± 1.3	6.9 ± 2	6.97 ± 9.02	6.27 ± 6.05	1.6 ± 1.4	2.6 ± 1.6	N/A	N/A	N/A	N/A	N/A	N/A
Holzer et al. [17]	N/A	N/A	N/A	N/A	N/A	N/A	N/A	N/A	N/A	N/A	N/A	N/A
Chang et al. [18]	N/A	N/A	N/A	N/A	N/A	N/A	N/A	N/A	N/A	N/A	N/A	N/A
Joseph et al. [19]	N/A	N/A	N/A	N/A	N/A	N/A	N/A	N/A	N/A	N/A	N/A	N/A
Kim et al. [20]	N/A	N/A	6.6 ± 1.9	10.05 ± 4.3	1.8 ± 0.9	2.4 ± 1	01	02	02	01	42	08
Kotani et al. [21]	3.5 ± 1.8	11.7 ± 3.9	7 ± 2.6	9 ± 4.6	N/A	N/A	N/A	N/A	N/A	N/A	N/A	N/A
Passerini et al. [22]	N/A	N/A	N/A	N/A	N/A	N/A	24	14	43	23	26	18
D’Silva et al. [23]	2 ± 1	3 ± 1	N/A	N/A	N/A	N/A	N/A	N/A	N/A	N/A	N/A	N/A
Ming et al. [24]	N/A	N/A	5.17 ± 1.61	6.7 ± 2.34	N/A	N/A	N/A	N/A	N/A	N/A	N/A	N/A
Alharbi et al. [25]	1.64 ± 1.41	3.58 ± 2.05	5.45 ± 2.14	7.99 ± 3.35	1.68 ± 0.29	1.61 ± 0.25	N/A	N/A	N/A	N/A	N/A	N/A
Kan et al. [26]	8.12 ± 2.01	16.01 ± 2.44	7.47 ± 2.6	7.07 ± 2.54	N/A	N/A	N/A	N/A	N/A	N/A	N/A	N/A
Ordás et al. [27]	4.57 ± 2.1	5.49 ± 1.13	7.55 ± 2.73	10.24 ± 5.42	N/A	N/A	04	02	15	05	16	16
Buhur et al. [28]	2.25 ± 0.8	3.84 ± 1.2	6.8 ± 0.16	6.9 ± 0.16	1.2 ± 0.22	2.24 ± 0.13	N/A	N/A	N/A	N/A	N/A	N/A
Total participants/total no. of events	968	1,025	1,075	1,328	327	550	252	156	189	109	189	109

Table 8 summarizes secondary outcomes across included studies comparing LM and OM. For continuous outcomes hospital stay, size of the largest myoma, and postoperative hemoglobin drop: data are presented as minimum, maximum, range, mean, and SD. For categorical outcomes miscarriage, VD, and CS after myomectomy: the number of events, relative risk (RR), and percentage occurrence were reported. Standardized mean differences (SMD) and *P*-values were included where applicable to reflect statistical significance. *N/A* indicates data not reported or not applicable.

**Table 8:** Summary of secondary outcomes data. Min, minimum; Max, maximum; SD, standard deviation; SMD, standard mean difference; OM, open myomectomy; LM, laparoscopic myomectomy; CS, cesarean section; VD, vaginal delivery

Outcome measured	No. of studies	No. of participants (LM)	No. of participants (OM)	Min (LM)	Min (OM)	Max (LM)	Max (OM)	Range (LM)	Range (OM)	Mean (LM)	Mean (OM)	SD (LM)	SD (OM)	No. of events (LM)	No. of events (OM)	RR	Percentage (LM)	Percentage (OM)	SMD	*P*-value
Hospital stay (days)	8	968	1,025	I.64	3	8.12	16.01	6.48	13.01	3.6	7.05	2.06	4.54	N/A	N/A	N/A	N/A	N/A	1.76	<0.00001
Size of the largest myoma (cm)	9	1,075	1.328	5.17	6.27	7.55	10.24	2.38	3.97	6.67	7.96	0.831	1.46	N/A	N/A	N/A	N/A	N/A	0.46	<0.00001
Hemoglobin drop after operation (g/dL)	5	327	550	1.2	2.17	1.8	2.6	0.6	0.43	1.48	2.35	0.26	0.19	N/A	N/A	N/A	N/A	N/A	1.44	0.04
Miscarriage after myomectomy	4	252	156	N/A	N/A		N/A		N/A		N/A		N/A	35	22	0.96	13.5	14.1	0.9	N/A
VD after myomectomy	4	189	109	N/A	N/A	N/A	N/A	N/A	N/A	N/A	N/A	N/A	N/A	67	35	1.18	37.9	32.1	0.27	N/A
CS after myomectomy	4	189	109	N/A	N/A	N/A	N/A	N/A	N/A	N/A	N/A	N/A	N/A	97	63	0.82	47.4	57.8	0.17	N/A

Table 9 summarizes the primary outcomes comparing LM and OM. Effect sizes, confidence intervals, and GRADE certainty ratings generated using GRADEpro software [33] are presented to reflect the strength and reliability of evidence across studies.

**Table 9:** Summary of findings tables (SOF) for primary outcomes generated from GRADEpro. LM compared to OM for women with uterine fibroids: Patient or population: women with uterine fibroids (18-50 years); intervention: LM; Comparison: OM; Setting: systematic review and meta-analysis Certainty ratings were generated using GRADEpro Guideline Development Tool [33]. The risk in the intervention group (and its 95% CI) is based on the assumed risk in the comparison group and the relative effect of the intervention. Symbols indicate confidence in the evidence: ⨁⨁⨁⨁ High, ⨁⨁⨁◯ Moderate, ⨁⨁◯◯ Low, ⨁◯◯◯ Very low. CI, confidence interval; RR, risk ratio; SMD, standardized mean difference; OM, open myomectomy; LM, laparoscopic myomectomy

Outcome; no. of participants (studies)	Relative effect (95% CI)	Anticipated absolute effects (95% CI)			Certainty	What happens
		Open Myomectomy	Laparoscopic Myomectomy	Difference		
Blood loss (mL); o. of participants: 1949 (LM = 962, OM = 987); 7 studies (6 non-RCT, 1 RCT)	-	Mean = 486.13 (SD 409.09) (Arm = Min-Max (Range)) OM = 79.22-1290.48 (1211.26) mL	Mean = 205.97 (SD 131.59) (Arm = Min-Max (Range)) LM = 44.4-406.6 (362.2)	SMD 1.21 lower (1.7 lower to 0.72 lower)	⨁⨁⨁◯ Moderate^a^	The meta-analysis of 7 studies indicates a significant reduction in blood loss in LM as compared to OM. The SMD-1.21 (-1.70, -0.72) shows a large effect size favoring LM. The high heterogeneity indicates study variation.
Duration of surgery (minutes); No. of participants: 2,917 (LM = 1,564, OM = 1,353); 11 studies (9 non-RCTs, 2 RCTs)	-	Mean = 98.55 (SD 19.35) Arm = Min-Max (Range) OM = 68-135.73 (67.73) minutes	Mean = 111.43 (SD = 34.56) Arm = Min-Max (Range) LM = 56.91-170.28 (113.3) minutes	MD 12.29 higher (6.66 lower to 31.23 higher)	⨁⨁⨁◯ Moderate^a^	The meta-analysis of 11 studies indicates that the MD in the duration of surgery is 12.29 minutes, suggesting a longer time for LM than open OM. However, it is not statistically significant. Test for overall effect: Z = 1.27 (P = 0.20). High heterogeneity (I = 97%) indicates substantial study variation.
Number of removed myomas (n), No. of participants: 2,683 (LM = 1,179, OM = 1,504), 8 studies (7-non-RCT, one RCT)	-	Mean = 3.34 (SD 1.31), OM (Arm = Min-Max) (Range), OM = 2.37-6.5 (4.13))-	Mean = 2.62 (SD 0.97), LM (Arm = Min-Max) (Range), LM = 1.5-4.18 (2.68)	SMD 0.29 lower (0.37 lower to 0.21 lower)	⨁⨁⨁◯ Moderate^b^	The meta-analysis of 8 studies indicates that the number of myomas removed by LM (MD -0.29) is slightly lower than OM. However, heterogeneity is a high I² value (71%), showing that there is considerable variability in the effect size. The overall effect is statistically significant, with a P-value of 0.0001
Pregnancy rate after myomectomy, No. of participants: 2,539 (LM = 1,367, OM = 1,172), 8 studies (7 non-RCT, 1 RCT)	RR 1.31 (1.07-1.62)	22.4%	29.3% (23.9-36.2)	6.9% more (1.6 more to 13.9 more)	⨁⨁⨁◯ Moderate^b^	The overall RR for pregnancy rate is 1.31 [1.07, 1.62], which suggests no significant difference in pregnancy rates between LM and OM, as the CI includes 1. The moderate heterogeneity indicates some variation among the study results

Table 10 presents secondary outcomes comparing LM and OM, including hospital stay, myoma size, hemoglobin drop, and reproductive outcomes. It summarizes relative effects, anticipated absolute differences, and GRADE certainty ratings across studies to assess clinical impact and evidence strength. 

**Table 10:** Summary of findings (SOF) for secondary outcomes generated from GRADEpro. The risk in the intervention group (and its 95% CI) is based on the assumed risk in the comparison group and the relative effect of the intervention (and its 95% CI). Source:  [33]. CI, confidence interval; MD, mean difference; RR, risk ratio; SMD, standardized mean difference; OM, open myomectomy; LM, laparoscopic myomectomy Symbols (GRADE certainty): ⨁⨁⨁⨁ High certainty ⨁⨁⨁◯ Moderate certainty ⨁⨁◯◯ Low certainty ⨁◯◯◯ Very low certainty Explanations: a. Retrospective study design introduces risk of bias. b. Data were collected from hospital records and analyzed retrospectively. c. Most included studies were retrospective; only two were RCTs. d. Retrospective design contributes substantially to the overall risk of bias.

Outcome: No. of participants (studies)	Relative effect (95% CI)	Anticipated absolute effects (95% CI)			Certainty	What happens
		Open myomectomy	Laparoscopic myomectomy	Difference		
Hospital stay (days): No. of participants: 1,993 (LM = 968, OM = 1025), 8 studies (7 non-RCT, one RCT)	-	Mean = 7.05 (SD 4.54) days, Arm = Min-Max (Range), OM = 6-16.01 (13.01) days	Mean = 3.6 (SD 2.06) Arm = Min-Max (Range), LM = 1.64-8.12 (6.48) days	SMD 1.76 lower (2.49 lower to 1.04 lower)	⨁⨁⨁◯ Moderate^a^	The meta-analysis of 8 studies demonstrates that the overall length of hospital stay in days is significantly shorter in LM (SMD-1.76) than in OM. High heterogeneity (I² = 97%) indicates substantial study variability in the effect size across the studies. The overall effect is statistically significant with a *P*-value of <0.0001.
The size of the largest myoma removed: No. of participants: 2,403 (LM = 1,075, OM = 1,328) 9 studies (8 non-RCT, 1 RCT)	-	Mean = 7.96 cm (SD 1.46) in OM Arm = Min-Max (Range), OM = 6.27-10.24 (3.97) cm	Mean = 6.67 cm (SD 0.831) LM Arm = Min-Max (Range) LM = 5.17-7.55 (2.38) cm	SMD 0.46 lower (0.55 lower to 0.38 lower)	⨁⨁⨁◯ Moderate^c^	The meta-analysis of 9 studies suggests that the size of myomas removed in LM is smaller than in OM (SMD -0.42). The overall effect is statistically significant (*P* =< 0.00001). Heterogeneity is I² = 85%.
Hemoglobin drop after operation: No. of participants: 877 (LM = 327, OM = 550), 5 studies (4 non-RCT, 1 RCT)	-	Mean = 2.35 (SD = 0.19) Arm = Min-Max (Range) OM = 2.17-2.6 (0.43)	Mean = 1.48 (SD 0.26) Arm = Min-Max (Range), LM = 1.2-1.8 (0.6) gm/dL	SMD 1.44 lower (2.81 lower to 0.07 lower)	⨁⨁⨁◯ Moderate^d^	The overall mean difference of -1.44 from 5 studies' data indicates that LM is statistically significantly associated with a reduction in hemoglobin level postoperatively as compared to OM. The *P*-value is 0.04.
Miscarriage after Myomectomy № of participants: 408 (LM=252, OM=156) 4 Studies non-RCT, 1 RCT)	RR 0.96 (0.49 to 1.86)	14.1%	13.5% (6.9 to 26.2)	0.6% fewer (7.2 fewer to 12.1 more)	⨁⨁⨁◯ Moderate^b^	The overall RR of 0.96 suggests no significant difference in miscarriage rates between LM and OM, as the 95% CI of 0.49 to 1.86 includes 1. The low to moderate heterogeneity (I² = 24%) indicates that the variability among the studies is not substantial, meaning the studies are relatively consistent in their findings
Vaginal delivery after Myomectomy: № of participants: 298 (LM=189, OM=109) 4 Studies non-RCT, 1 RCT)	RR 1.18 (0.88 to 1.57)	32.1%	37.9% (28.3 to 50.4)	5.8% more (3.9 fewer to 18.3 more)	⨁⨁⨁◯ Moderate^a^	The overall RR of 1.18 suggests a trend towards a higher rate of vaginal delivery with LM compared to OM. Still, this finding is not statistically significant as the 95% CI of 0.88 to 1.57 includes 1. The lack of heterogeneity (I² = 0%) indicates that the studies are consistent in their findings.
Caesarean section after Myomectomy № of participants: 298 (LM=189, OM=109) 4 Studies non-RCT, 1 RCT)	RR 0.82 (0.62 to 1.08)	57.8%	47.4% (35.8 to 62.4)	10.4% fewer (22 fewer to 4.6 more)	⨁⨁⨁◯ Moderate^c^	The overall RR of 0.82 suggests a trend towards a lower rate of CS with LM compared to OM, but this finding is not statistically significant as the 95% CI of 0.62 to 1.08 includes 1. The moderate heterogeneity (I² = 33%) indicates that the variability among the studies is present but not substantial

Data synthesis/outcomes analysis and descriptive statistics

Meta-analysis of Primary Outcomes From Review Manager (RevMan) Version 5.4.1

**Figure 6:**
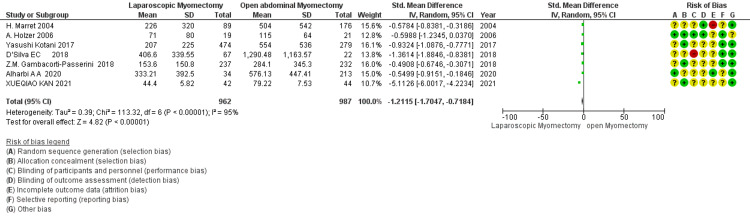
Forest plot comparing blood loss (mL) between LM and OM. OM, open myomectomy; LM, laparoscopic myomectomy

Blood loss (mL): LM vs. OM: The meta-analysis shows that blood loss is less in LM than in OM (Figure 6). There is a statistically significant (*P* < 0.00001) difference with the overall effect favoring LM over OM in terms of the standard mean difference. High heterogeneity (I² = 95%) suggests variability in study results [34].

**Figure 7:**
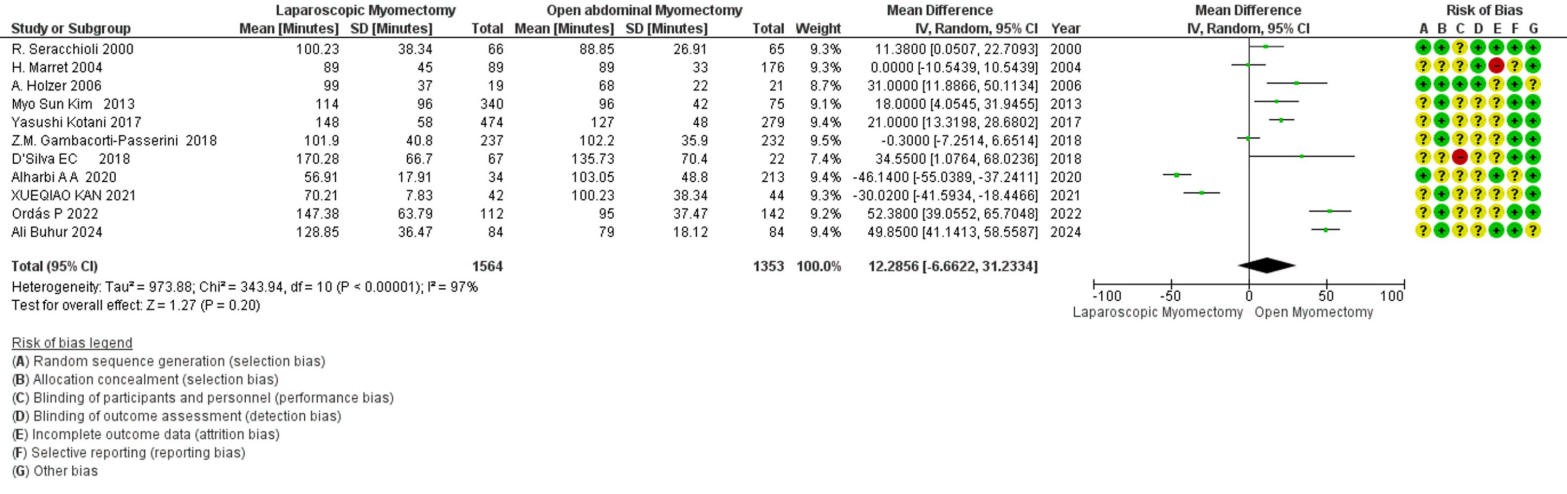
Forest plot: duration of surgery (minutes).

Duration of surgery (min): The meta-analysis of studies demonstrates that the mean difference in the duration of surgery is 12.29 minutes, suggesting a longer time for LM than OM (Figure 7). However, it is not statistically significant (*P *= 0.20). High heterogeneity (I² = 97%) indicates substantial study variation.

**Figure 8:**
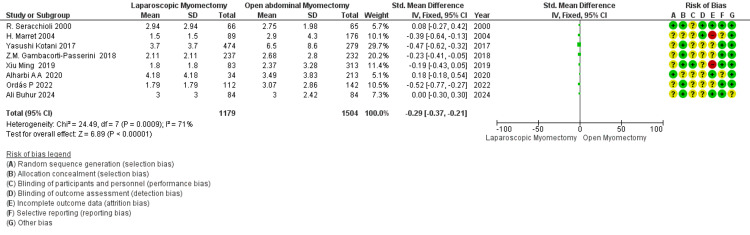
Forest plot: number of removed myomas (n).

Number of removed myomas (*n*): Data from 8 studies indicate that the number of myomas removed by LM (SMD - 0.22) is fewer than OM (Figure 8). However, heterogeneity is a high I² value (71%), showing that there is considerable variability in the effect size. The overall effect is statistically significant, with a *P*-value of 0.01.

**Figure 9:**
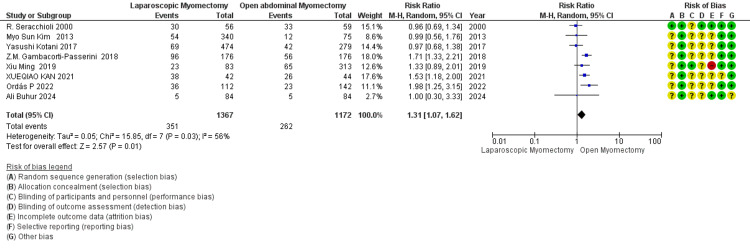
Forest plot: pregnancy rate after myomectomy.

Pregnancy rate after myomectomy: The analysis of 8 studies indicates that LM has slightly higher odds of pregnancy events than OM (Figure 9). The overall RR for pregnancy rate is around 1.31 with a CI of 1.07 to 1.62, which suggests no significant difference in pregnancy rates between LM and OM, as the CI includes 1. The moderate heterogeneity indicates some variation among the study results.

Meta-analysis of Secondary Outcomes From Review Manager (RevMan) Version 5.4.1

**Figure 10:**
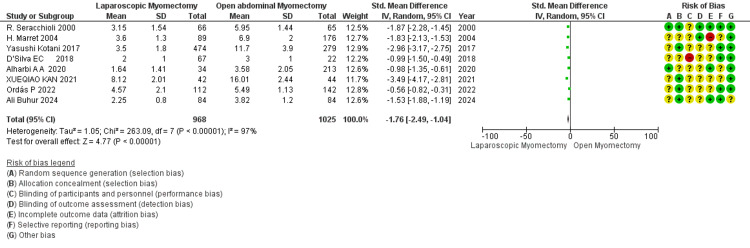
Forest plot: hospital stay (days).

Hospital stay (days): LM vs. OM: The overall length of hospital stay in days is significantly shorter in LM (SMD - 1.76) than in OM (Figure 10). High heterogeneity (I² = 97%) indicates substantial study variability in the effect size across the studies. The overall effect is highly statistically significant, with a *P*-value of <0.0001 [34].

**Figure 11:**
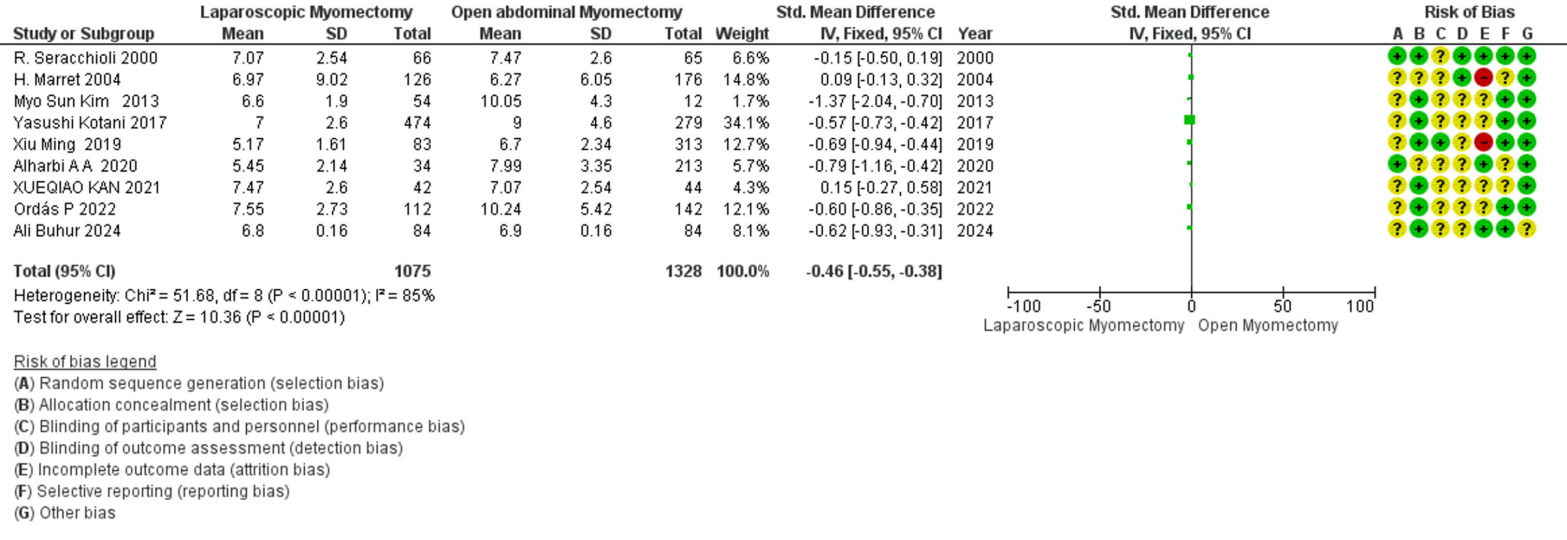
Forest plot: the size of the largest myoma removed (cm).

The size of the largest myoma removed (cm): The meta-analysis of 9 studies suggests that the size of myomas removed in LM is smaller than in OM (SMD - 0.46) (Figure 11). The overall effect is statistically significant (*P *=< 0.00001). Heterogeneity is I² = 85%.

**Figure 12:**
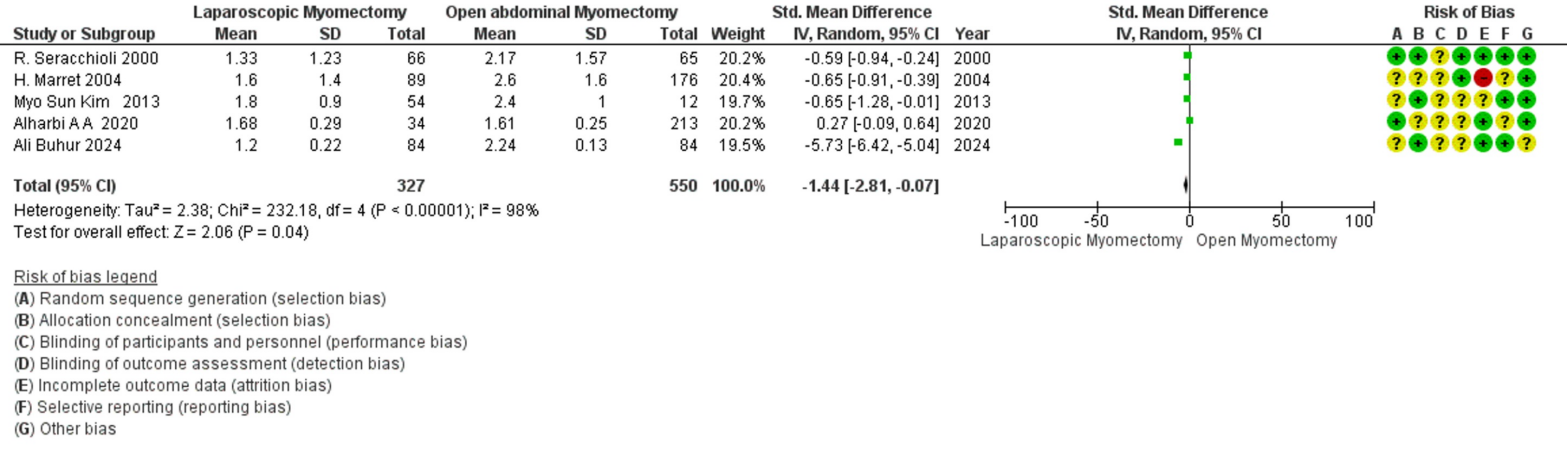
Forest plot: Hb drop after operation (g/dL).

Hb drop after operation (gm/dL): The overall mean difference of -1.44 from 5 studies' data indicates that LM is statistically significantly associated with a reduction in hemoglobin level postoperatively as compared to OM (*P*-value = 0.04) (Figure 12).

**Figure 13:**
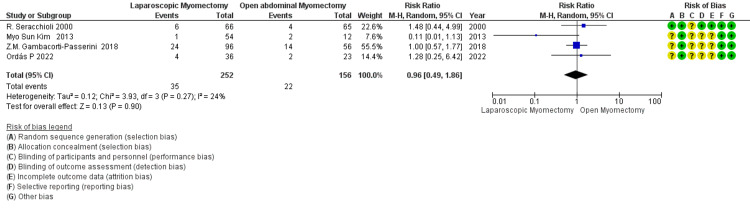
Forest plot: miscarriage after myomectomy.

Miscarriage after myomectomy: The overall RR of 0.96 suggests no significant difference in miscarriage rates between LM and OM, as the 95% CI of 0.49 to 1.86 includes 1 (Figure 13). The low to moderate heterogeneity (I² = 24%) indicates that the variability among the studies is not substantial, meaning the studies are relatively consistent in their findings.

**Figure 14:**
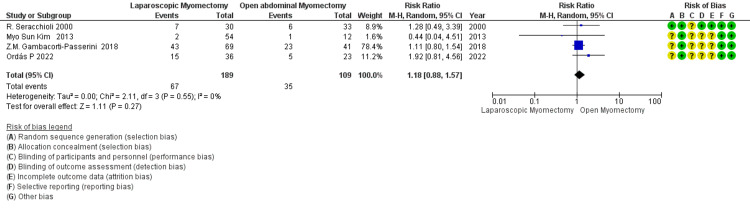
Forest plot: vaginal delivery (VD) after myomectomy.

VD after myomectomy: The overall RR of 1.18 suggests a trend toward a higher rate of VD with LM compared to OM (Figure 14). Still, this finding is not statistically significant as the 95% CI of 0.88 to 1.57 includes 1. The lack of heterogeneity (I² = 0%) indicates that the studies are consistent in their findings regarding VD rates after myomectomy.

**Figure 15:**
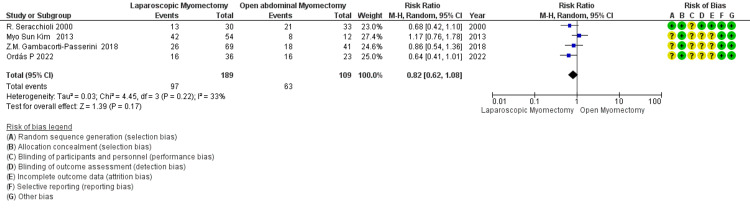
Forest plot of cesarean section (CS) after myomectomy.

CS after myomectomy: The overall RR of 0.82 suggests a trend toward a lower rate of CS with LM than OM (Figure 15). Still, this finding is not statistically significant as the 95% CI of 0.62 to 1.08 includes 1. The moderate heterogeneity (I² = 33%) indicates that the variability among the studies is present but not substantial.

Funnel Plots for Publication Bias Generated in Review Manager (RevMan) version 5.4.1

Primary outcomes summary: Funnel plots for all four primary outcomes-blood loss, duration of surgery, number of removed myomas, and pregnancy rate-show relatively symmetrical distributions around their respective pooled effect estimates, suggesting minimal evidence of publication bias [34]. For pregnancy rate, the pooled RR was 1.31 (95% CI: 1.07-1.62), indicating a statistically significant advantage for LM. Despite high heterogeneity in blood loss (I² = 95%) and duration of surgery (I² = 97%), the symmetry of the plots supports the credibility of the meta-analytic findings.

Secondary outcomes summary: Funnel plots for secondary outcomes, including hospital stay, size of the largest myoma removed, postoperative hemoglobin drop, VD, and CS, show symmetrical or relatively symmetrical distributions around their respective pooled effect estimates or null lines, indicating minimal evidence of publication bias. In contrast, the funnel plot for miscarriage after myomectomy displays asymmetry, suggesting potential publication bias or heterogeneity among the included studies.

Results summary 

The analysis revealed a statistically significant reduction in intraoperative blood loss in the LM group compared to OM (SMD = -1.21), consistent with the minimally invasive nature of laparoscopy and its reduced tissue trauma. Postoperative hemoglobin drop was also significantly lower in LM (SMD = 1.44), reinforcing the clinical benefit of reduced blood loss. The length of hospital stay was significantly shorter in LM than OM (SMD = -1.76), supporting faster recovery and earlier return to normal activities. Operative time was longer in LM (MD = 12.29 minutes; *P* = 0.20), reflecting the technical complexity of the procedure, although this difference was not statistically significant and may not be clinically impactful. The number and size of myomas removed were slightly lower in LM, but these differences were not statistically significant (P > 0.05), suggesting that LM may be less suitable for cases involving multiple or larger fibroids.

Regarding fertility outcomes, the pregnancy rate was slightly higher in LM than OM (29.3% vs. 22.4%; RR = 1.31; *P* = 0.01), but this difference was not statistically significant, indicating no clear clinical advantage. VD rates after myomectomy showed a trend toward LM (37.9% vs. 32.1%; RR = 1.18; *P* = 0.27), while CS rates were lower in LM (47.4% vs. 57.8%; RR = 0.82; *P* = 0.17); however, both findings lacked statistical significance. Miscarriage rates were comparable between LM and OM (13.5% vs. 14.1%; RR = 0.96; *P* = 0.90), indicating no significant difference. Overall, while LM demonstrates several clinically favorable trends, not all outcomes reached statistical significance, and its suitability may depend on individual patient factors such as fibroid size and number.

Future recommendations

More RCTs are needed to evaluate LM and OM across varying fibroid sizes and numbers, as well as their effects on future pregnancy and fertility outcomes. Standardized patient selection guidelines and increased availability of skilled surgeons are essential. Longitudinal studies should assess long-term fertility outcomes and morbidity, including recurrence rate, uterine rupture risk, repeat myomectomy, and future hysterectomy risk.

Limitations

Many of the included studies were retrospective, which increases the risk of bias. Significant heterogeneity in patient characteristics and surgical techniques further limits the ability to draw definitive conclusions regarding clinical and fertility outcomes between LM and OM.

## Conclusions

UFs are common benign neoplasms of the uterus, primarily affecting women of reproductive age. Although often asymptomatic, approximately 30% of patients experience symptoms, with abnormal uterine bleeding being the most common. Clinical features vary based on fibroid size, location, and number. Myomectomy remains a uterine-preserving surgical option, especially for women seeking to conceive, with LM and OM being the most commonly performed procedures. LM was associated with significantly reduced intraoperative blood loss, lower postoperative hemoglobin drop, and shorter hospital stays, supporting its role in promoting early recovery. However, LM had a longer operative time, reflecting its technical complexity. The number and size of fibroids removed were slightly lower with LM than OM, with statistically significant differences, though high heterogeneity across studies suggests variability in outcomes. These findings indicate that LM may be less suitable for multiple or larger fibroids. Obstetric outcomes showed a slightly higher pregnancy rate in LM compared to OM, though not statistically significant. Vaginal delivery rates were higher with LM, and caesarean section rates were lower; however, both findings lacked statistical significance. Miscarriage rates were comparable between LM and OM.

OM has traditionally been preferred for multiple fibroids, yet the benefits of LM remain under debate. For women wishing to conceive, a thorough evaluation of fibroid characteristics and their impact on the uterine cavity is essential. Surgical decisions should consider factors such as fibroid size and number, uterine incision site, diathermy use, suturing technique, and surgeon expertise. LM offers advantages, including reduced blood loss, shorter hospital stays, and faster recovery, but requires longer operative time and advanced surgical skill. It is most effective for fibroids fewer than four and under 10 cm in size, while OM is generally preferred for larger or multiple fibroids. Obstetric outcomes after LM are promising but not statistically superior. Future risks such as uterine dehiscence depend on surgical technique and fibroid characteristics, emphasizing the importance of careful patient selection and surgical expertise.
